# Hypoxia-induced CCL28 promotes recruitment of regulatory T cells and tumor growth in liver cancer

**DOI:** 10.18632/oncotarget.12409

**Published:** 2016-10-03

**Authors:** Li Ren, Yang Yu, Li Wang, Zhifeng Zhu, Rong Lu, Zhi Yao

**Affiliations:** ^1^ Department of Clinical Laboratory, Tianjin Medical University Cancer Institute and Hospital, National Clinical Research Center for Cancer, Key Laboratory of Cancer Prevention and Therapy, Tianjin's Clinical Research Center for Cancer, Tianjin, P.R. China; ^2^ Department of Immunology, Tianjin Key Laboratory of Cellular and Molecular Immunology, Key Laboratory of Educational Ministry of China, School of Medical Sciences, Tianjin Medical University, Tianjin, P.R. China

**Keywords:** CCL28, Treg, HCC, angiogenesis, HIF1α

## Abstract

Tumor cells craft microenvironment to overcome growth disadvantages and adjust to escape the immunosurveillance during tumorigenesis and metastasis. The evolving adaption to the changing microenvironment is exemplified by the development of strategies to deal with hypoxia resulted from fast proliferation of the tumor cells. In this study, we found that hypoxia hepatocellular carcinoma (HCC) cells recruited Regulatory T cells (Tregs) and expressed more Chemokine (C-C motif) ligand 28 (CCL28). Deletion of CCL28 inhibited Treg recruitment. Furthermore, overexpression of CCL28 promoted tumor growth and Treg infiltration *in vivo*. Enhanced angiogenesis and VEGF expression was also observed. Moreover, inhibition of HIF1α reversed hypoxia-induced CCL28 upregulation. Taken together, our results demonstrate that HCC recruits Tregs to promote angiogenesis under hypoxic condition by upregulating CCL28 expression. These findings establish a link between Tregs and hypoxia in HCC growth and may provide a new potential therapeutic target for treating HCC.

## INTRODUCTION

During cancer development, tumor cells have to develop strategies to evade surveillance imposed by the host immune system, and to craft optimal microenvironment to promote their survival and growth in the body [1–5]. As one of such strategies, tumor cells may suppress the functions of effector T-cells by enlisting Treg cells to the tumor sites [6–9]. A previous study has suggested that liver inflammation is associated with recruitment of regulatory T cells [10]. The chemokine CC-chemokine ligand 28 (CCL28) production is also linked to this process. In ovarian cancer, CCL28 has been suggested to mediate the recruitment of the Treg cells to the tumor site [11]. However, it is not clear whether the Treg cells play the same critical role in escaping the host immune surveillance in liver cancer. By far, the mechanisms by which the Treg cells are recruited to the tumor site and promote tumor growth remain to be revealed for various cancers. Furthermore, within the disadvantaged hypoxic microenvironment caused by fast tumor cell proliferation, how pro-tumor growth is achieved in liver cancer is of great interests.

Hypoxia promotes tumor growth and increases risks of metastasis, and is closely linked to poor patient outcomes, which may be caused by various physiological and biochemical adaptations in the hypoxic tumors [12–14]. Among these changes, interactions between the chemokines and their receptors are significantly important [15]. Chemokines are secreted small peptides that can induce migration of leukocytes, and subject to the direct regulation by hypoxia inducible factor 1α (HIF1α) and have been suggested to play an important role in tumor angiogenesis and metastasis in various cancers [16–21]. Given the feature that chemokines and their receptors can promote the mobilization of various cell types in the body, it is likely that they link the hypoxic tumor microenvironment and the recruitment of the regulatory T cells to the tumor sites. However, except for ovarian cancer, this notion is yet to be confirmed in other cancers including the liver cancer.

In the current research, by generating a mouse model of liver cancer and employing an *in vivo* live imaging study on the mice, we aim at confirming if recruitment of regulatory T cells to the hypoxic hepatic tumors accounts for accelerated tumor growth *in vivo*, and identifying the molecular mechanisms governing this process. Specifically, we demonstrate that tumor hypoxia can induce elevated expression of CCL28 through HIF1α. Regulatory T cells can modulate the immune response and angiogenesis, resulting in effective immune escape and accelerated tumor growth *in vivo*.

## RESULTS

### Hypoxic hepatocellular carcinoma cells recruit Tregs by upregulating CCL28 *in vitro*

**Figure 1:**
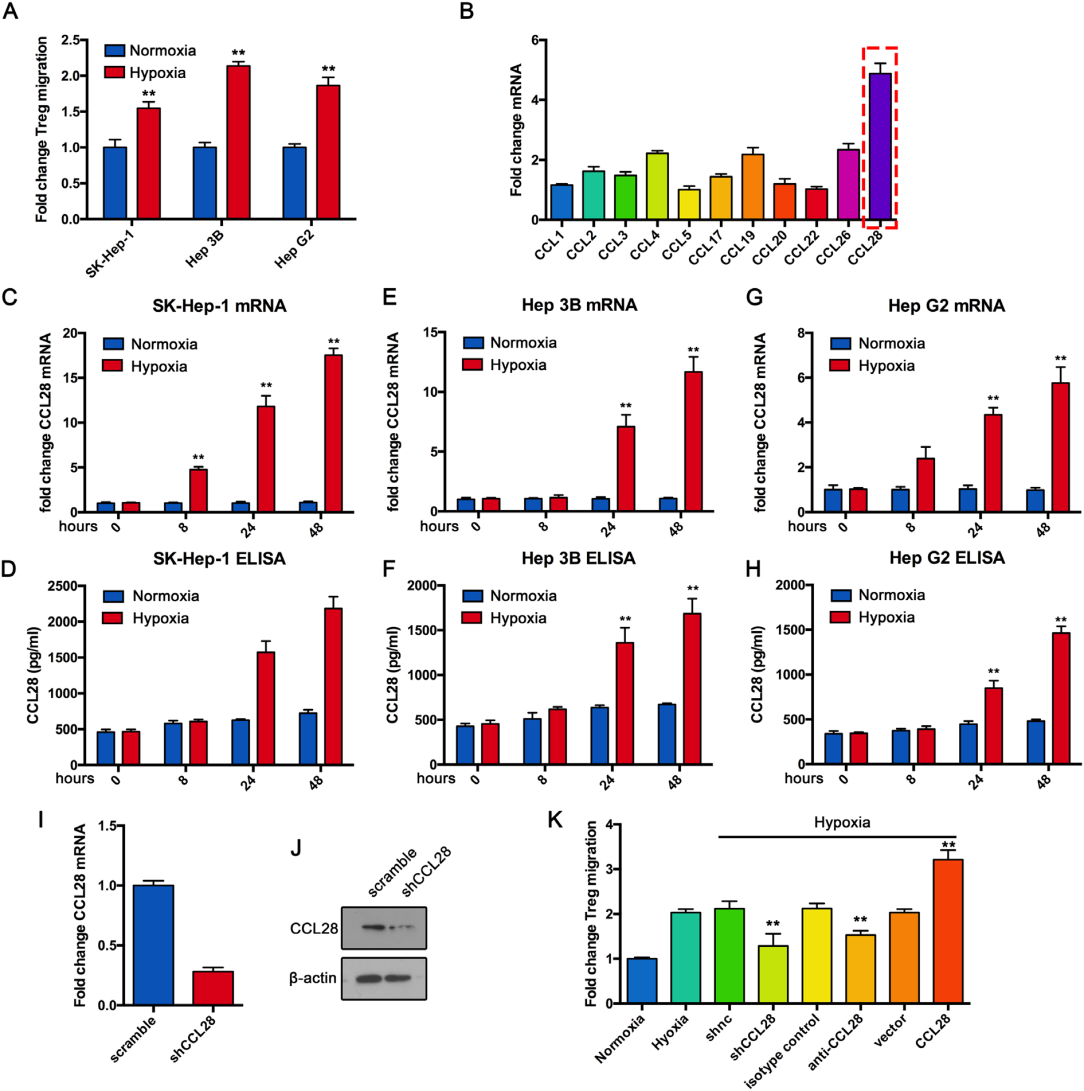
Hypoxic hepatocellular carcinoma cells recruit Tregs by upregulating CCL28 *in vitro* **A.** Treg cells were isolated from PBMCs of healthy adults and Transwell assay was performed for their migration toward supernatant from SK-Hep-1, Hep 3B and Hep G2 cells cultured under normoxic or hypoxic condition for 48 hours. **B.** qPCR analysis of indicated mRNA expression in SK-Hep-1 cells under hypoxic condition. qPCR and ELISA analysis of CCL28 expression in SK-Hep-1 **C.** and **D.**, Hep 3B **E.** and **F.** and Hep G2 **G.** and **H.** cells under normoxic or hypoxic condition. **I.** and **J.** CCL28 knockdown efficiency in SK-Hep-1 cells was analyzed by qPCR and western blot assay. **K.** Treg migration assay under indicated conditions. The data are shown as the means ± SD. *, P < 0.05, * *, P < 0.01.

To investigate whether hypoxic status could affect Treg recruitment by HCC cells, Treg migration assay was performed. Human peripheral blood mononuclear cells (PBMCs) from healthy donors were added to upper chamber of each insert. Then, supernatant of hypoxic or normoxic HCC cells (SK-Hep-1, Hep 3B and Hep G2) was added to lower chambers. Results showed that supernatant of hypoxic HCC cells significantly increased Tregs (CD4+CD25+FOXP3+) migration compared with controls (Figure 1A). Because CC chemokines are reported involved in Treg recruitment, we examined CC chemokines mRNA expression in hypoxic SK-Hep-1 cells. Results showed that CCL28 expression was significant increased under hypoxic status (Figure 1B). Similar results were obtained from SK-hep-1, Hep 3B and Hep G2 cells (Figure 1C, 1D and 1E). Data from ELISA confirmed the hypoxia-induced upregulation of CCL28 in supernatant of the three cell lines (Figure 1F, 1G and 1H).

To elucidate the role of CCL28 in hypoxia-induced Treg recruitment, we knockdowned CCL28 expression in SK-Hep-1 cells by siRNA (Figure 1I and 1J). Then Treg migration assay was performed. Results showed that CCL28 knockdown reversed hypoxia-induced Treg recruitment (Figure 1K). We also blocked CCL28 interaction with Tregs by anti-CCL28 antibody. Similar results were obtained (Figure 1K). To further confirmed the results, we overexpressed CCL28 in SK-Hep-1 cells. Treg migration assay showed that CCL28 overexpression enhanced hypoxia-induced Treg recruitment (Figure 1K). Taken together, these data demonstrate that hypoxia induces HCC cells recruit Tregs by upregulating CCL28 expression.

### CCL28 promotes tumor formation *in vivo*

**Figure 2:**
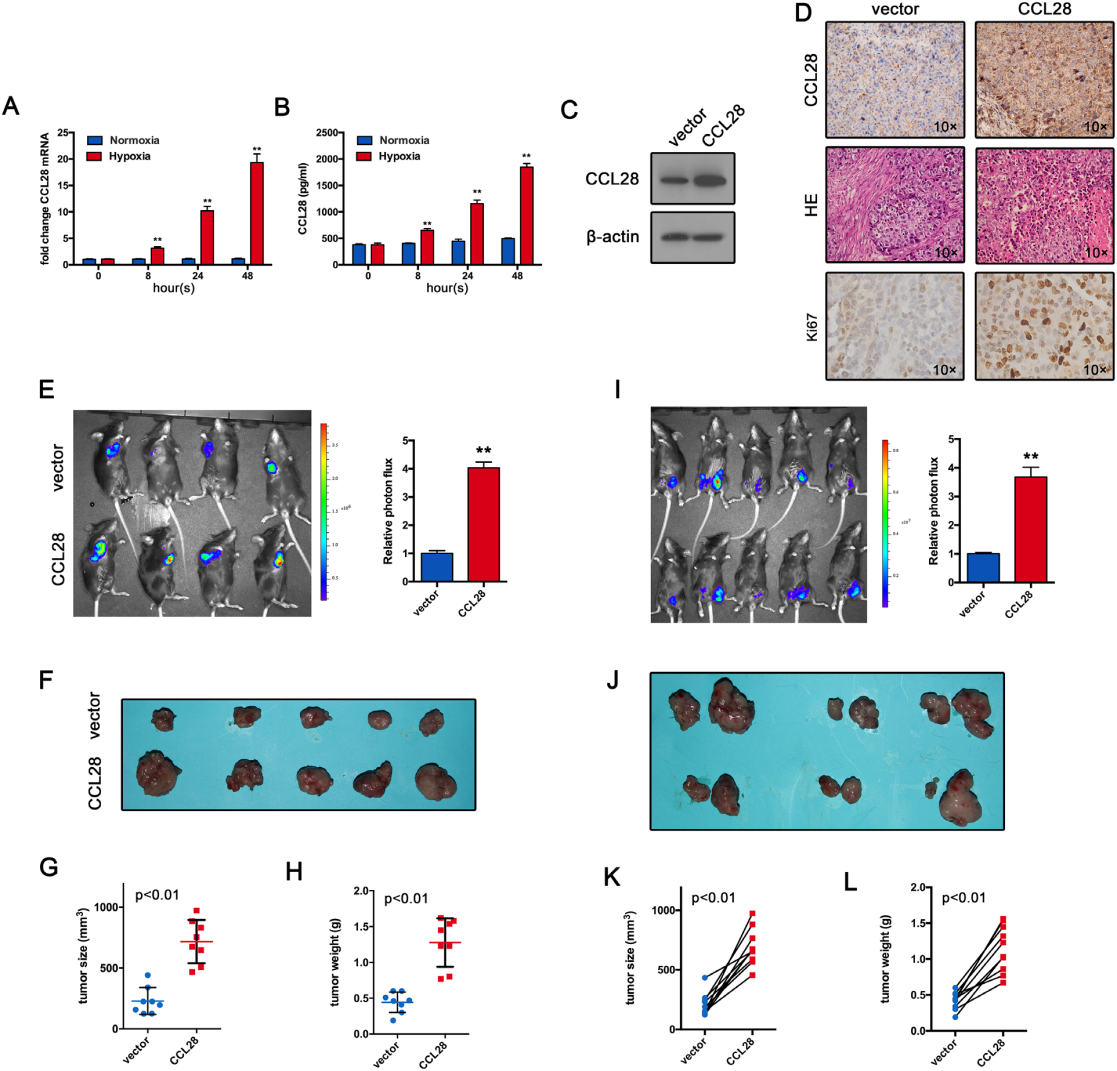
CCL28 promotes tumor formation *in vivo* **A.** and **B.** qPCR and ELISA analysis of CCL28 expression Hepa1-6 cells under normoxic or hypoxic condition. **C.** CCL28 overexpression efficiency in Hepa1-6 cells was analyzed by western blot. **D.** Tumor sections(Hepa1-6) were stained with anti-CCL28 antibody or Ki-67 antibody. **E.** Hepa1-6 cells infected with CCL28 expressing vector or control cells were injected to C57BL/6 mice around the right flanks subcutaneously. At day 21, *in vivo* bioluminescence imaging was performed. **F**, **G.** and **H.** Tumor size and weight were measured. **I.** CCL28-Hepa1-6-luci cells and the Control-Hepa1-6-luci cells were implanted around the left- and right-tibia, respectively, of the same mouse. At day 21, *in vivo* bioluminescence imaging was performed. **J, K.** and **L.** Tumor size and weight were measured. The data are shown as the means ± SD. *, P < 0.05, * *, P < 0.01.

Given the correlation of high level CCL28 and hypoxia, we further hypothesized that CCL28 might promote *in vivo* growth of HCC cells due to the highly hypoxic microenvironment in tumor. We first examined if hypoxia-induced CCL28 upregulation also work in a mouse hepatoma cell line (Hepa1-6), results showed hypoxia induced an increase of CCL28 expression (Figure 2A and 2B). Then we overexpressed CCL28 in Hepa1-6 cells (Figure 2C and 2D), together with a luciferase reporter gene (luc2). The resultant cell line was named CCL28-Hepa1-6-luci. Then Hepa1-6 cells infected with CCL28 expressing vector or control cells were injected to C57BL/6 mice around the right flanks subcutaneously. At day 21, *in vivo* bioluminescence imaging was performed. As shown in Figure 2E, CCL28 overexpression resulted in significant stronger photon signals than controls. Accordingly, the volume and weight of CCL28-Hepa1-6-luci tumors were higher than those of Control-Hepa1-6-luci ones (Figure 2F, 2G and 2H). And CCL28 overexpression tumor exhibited higher Ki67 positive rate (Figure 2D). Therefore, the current data suggest that CCL28 upregulation promotes HCC tumor growth *in vivo*.

The difference of immunoprotection among individuals may account for the difference in the *in vivo* tumor growth. To exclude this possibility, we implanted the CCL28-Hepa1-6-luci cells and the Control-Hepa1-6-luci cells around the right- and left-tibia, respectively, of the same mouse. At day 21, *in vivo* bioluminescence imaging was performed. In consistent with previous data, the CCL28-Hepa1-6-luci tumors have stronger photon signals than the control-Hepa1-6-luci tumors (Figure 2I). the volume and weight of CCL28-Hepa1-6-luci tumors were higher than those of control-Hepa1-6-luci ones (Figure 2J, 2K and 2L). These results, together with previous data, provide strong evidences that CCL28 promote tumor formation of HCC cells *in vivo*.

### CCL28 recruits Tregs *in vivo*

**Figure 3:**
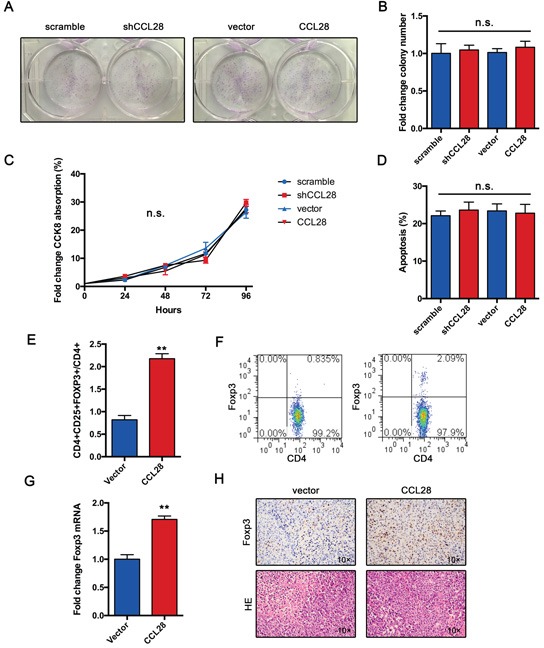
CCL28 recruits Tregs *in vivo* **A.** and **B.** Colony formation assay of Hepa1-6 cells infected with CCL28 expressing lentivirus or control. **C.** Proliferation curve of Hepa1-6 cells infected with CCL28 expressing lentivirus or control. **D.** Rate of cisplatin-induced apoptosis of Hepa1-6 cells infected with CCL28 expressing lentivirus or control. **E.** and **F.** Frequency of Tregs in Hepa1-6 or Hepa1-6-CCL28 tumors. **G.** mRNA expression of FOXP3 in Hepa1-6 or Hepa1-6-CCL28 tumors. **H.** IHC staining of FOXP3 in Hepa1-6 or Hepa1-6-CCL28 tumors. The data are shown as the means ± SD. *, P < 0.05, * *, P < 0.01. n.s., no significant.

To functionally validate the role of CCL28 in HCC, we performed colony formation assay. Results showed that either CCL28 knockdown or overexpression has no impact on the capacity of colony formation (Figure 3A and 3B). Cell proliferation was determined by CCK8 assay. In agreement with previous results, either CCL28 knockdown or overexpression did not affect Hepa1-6 cell proliferation (Figure 3C). Furthermore, we examined if CCL28 regulates cisplatin-induced apoptosis. Data showed that alteration of CCL28 expression did not affect cisplatin-induced apoptosis (Figure 3D). These data indicate that CCL28 exert its oncogenic function in a non–cell autonomous way.

*In vitro* study indicate that hypoxic HCC cells recruit Tregs by CCL28. We wonder if CCL28 exert same function *in vivo*. As we expected, flow cytometry analysis showed that the frequency of Tregs in tumor tissues was increased in CCL28 expression group (Figure 3E and 3F). The expression of FOXP3 mRNA was also upregulated in CCL28 expression group (Figure 3G). Immunochemistry analysis showed that frequency of FOPX3+ cells was higher in CCL28 expression group (Figure 3H). Taken together, these results indicate that CCL28 exert its oncogenic role in a non–cell autonomous way and probably by recruiting Tregs.

### CCL28 enhances Treg-induced angiogenesis

**Figure 4:**
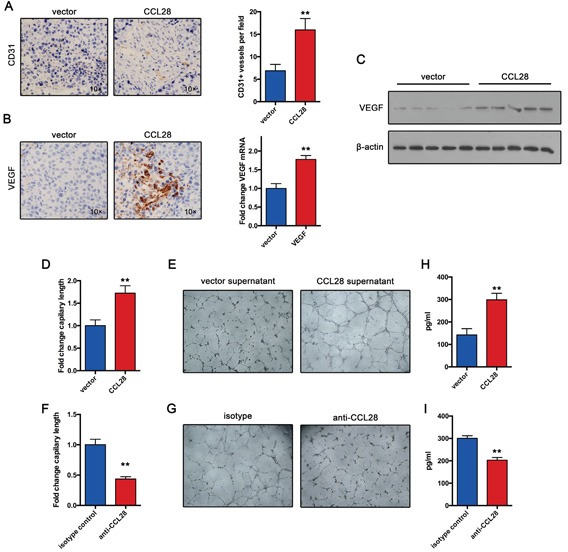
CCL28 enhances Treg-induced angiogenesis **A.** Vasculature density in Hepa1-6 and Hepa1-6-CCL28 tumors determined by immunohistochemistry. **B.** VEGF expression in Hepa1-6 and Hepa1-6-CCL28 tumors analyzed by immunohistochemistry or RT-qPCR assay. **C.** western blot analysis of VEGF expression in Hepa1-6 and Hepa1-6-CCL28 tumors. **D.** and **E.** Tregs incubated with CCL28 or vehicle for 12 hours. Then tube formation assay was performed. **F.** and **G.** Tregs incubated with anti-CCL28 antibody or isotype control for 12 hours. Then tube formation assay was performed. **H.** and **I.** VEGF expression in supernatant from Tregs treated with CCL28, vehicle, anti-CCL28 antibody or isotype control. The data are shown as the means ± SD. *, P < 0.05, * *, P < 0.01.

Previous study demonstrate that Tregs induce angiogenesis in ovarian cancer. So we speculated that CCL28 may exert its oncogenic function by promoting angiogenesis in HCC. To test this hypothesis, we examined CD31+ vessels in Hepa1-6 tumors. Results showed that CCL28 expression group produced more vessels in tumor tissue compared with control group (Figure 4A). Furthermore, we also examined VEGF expression in Hepa1-6 tumors. In consistent with angiogenesis results, CCL28 expression group expressed more VEGF in Hepa1-6 tumors (Figure 4B). Western blot assay confirmed the expression of VEGF in Hepa1-6 tumors (Figure 4C).

To further confirmed the role of CCL28 in Treg-induced angiogenesis, we incubated Tregs with CCL28 or vehicle for 12 hours. Then Tube formation assay was performed. Supernatant from CCL28 treated Tregs induced larger expansion of human umbilical vein endothelial cells, compared with controls (Figure 4D and 4E). In contrast, when we used anti-CCL28 antibody blocking CCL28 function, the results reversed (Figure 4F and 4G). Furthermore, we examined VEGF expression in supernatant from Tregs treated with CCL28, vehicle, anti-CCL28 antibody or isotype control. Results showed that CCL28 increased Treg expression of VEGF, and anti-CCL28 antibody decreased VEGF expression (Figure 4H and 4I). Taken together, these results indicate that CCL28 positive regulates angiogenesis in HCC by increasing Treg expression of VEGF.

### Hypoxia induced CCL28 upregulation is HIF1α dependent

**Figure 5:**
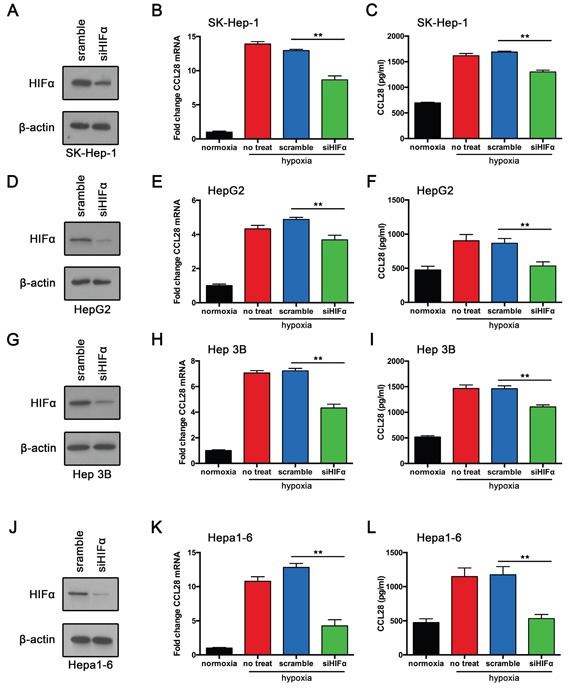
Hypoxia induced CCL28 upregulation is HIF1α dependent **A.** SK-Hep-1 cells transfected with HIF1α-siRNA (siHIF1α) or scramble control. Then western blot assay was performed. **B.** and **C.** RT-qPCR and ELISA analysis of CCL28 expression in SK-Hep-1 cells transfected with HIF1α-siRNA (siHIF1α) or scramble control. **D.** HepG2 cells transfected with HIF1α-siRNA (siHIF1α) or scramble control. Then western blot assay was performed. **E.** and **F.** RT-qPCR and ELISA analysis of CCL28 expression in HepG2 cells transfected with HIF1α-siRNA (siHIF1α) or scramble control. **G.** Hep 3B cells transfected with HIF1α-siRNA (siHIF1α) or scramble control. Then western blot assay was performed. **H.** and **I.** RT-qPCR and ELISA analysis of CCL28 expression in Hep 3B cells transfected with HIF1α-siRNA (siHIF1α) or scramble control. **J.** Hepa1-6 cells transfected with HIF1α-siRNA (siHIF1α) or scramble control. Then western blot assay was performed. **K.** and **L.** RT-qPCR and ELISA analysis of CCL28 expression in Hepa1-6 cells transfected with HIF1α-siRNA (siHIF1α) or scramble control. The data are shown as the means ± SD. *, P < 0.05, * *, P < 0.01.

Hypoxia-inducible transcription factors 1α plays a vital role in cancer response to hypoxic status. We speculated that HIF1α may participate in hypoxia-induced CCL28 upregulation and Treg recruitment. We knockdown HIF1α expression in HCC cell lines. Both qPCR assay and ELISA showed that HIF1α downregulation reversed hypoxia induced CCL28 upregulation in SK-Hep-1 cells (Figure 5). Similar results were also obtained from HepG2 and Hep3B cells. Furthermore, we knockdowned HIF1α in mouse hepatoma cell line (Hepa1-6). Results showed that HIF1α downregulation reversed hypoxia induced CCL28 upregulation (Figure 5). Together these results indicate that Hypoxia induced CCL28 upregulation is HIF1α dependent.

## DISCUSSION

In this study, we revealed the regulation of hypoxia on the Treg recruitment of hepatic tumor, which drives the *in vivo* survival and growth of the tumors. Specifically, proliferative tumor growth results in disadvantaged hypoxic microenvironment that is detrimental to tumor survival. The liver tumors may evade the host immunosurveillance and maintain the growth advantages by upregulating expression of CCL28 during hypoxia; and by binding to its receptor, CCR10, CCL28 can effectively recruit CCR10+ Treg cells to the tumor site, where this specific population of Treg cells repress the functions of effector T-cells, promoting the *in vivo* tumor growth. We have provided evidence that CCL28 is subject to specific regulation of hypoxia: CCL28 mRNA and protein levels were elevated in multiple hepatic tumor cell lines when cultured in hypoxic condition (Figure 1); and specific knockdown of HIF1α can reverse this process that has been observed for the hypoxic control/mock knockdown cells (Figure 5). Significantly, we observed an acceleration in tumor growth when a cell clone that overexpressed CCL28 was injected and allowed to grow in mice, and such growing advantages were mainly due to CCL28 overexpression and independent of immunoprotective differences among individuals (Figure 2). Interestingly, in the *in vitro* migration assay, the hypoxic culture supernatants could attract more CD4+CD25+FOXP3+ Treg cells than normoxic supernatants. Furthermore, the recruitment of the Treg cells can be effectively blocked by anti-CCL28 antibodies, suggesting CCL28 may enrich the Treg cells. We also confirmed that in the *in vivo* hepatic tumor model, foxp3 expression was upregulated in the tumor-infiltrated lymphocytes, providing evidence that the growth advantages enjoyed by the CCL28-overexpressing tumor cells may be mediated by the same mechanism as what we have observed for the *in vitro* recruitment of the CD4+CD25+FOXP3+ Treg cells. Therefore, in this study, we first demonstrate and confirm the link between hypoxia, chemokine, and the angiogenesis in liver cancer. Such regulatory network can provide new therapeutic targets in the liver cancer treatment, and can further assist with the understanding of immunoregulation of liver tumor development within disadvantaged tumor microenvironment.

We have provided evidence that liver tumor cells overcome disadvantaged hypoxic microenvironment by upregulating expression of CCL28 to recruit a specific Treg population at the tumor site. Although similar mechanism has been suggested in ovarian cancer [11], it has remained to be demonstrated in liver cancer. Particularly, there has been few studies on chemokines and their roles in tumor microenvironment crafting during hepatic tumorigenesis. We have confirmed that a similar strategy may have evolved in hepatic tumors where hypoxic microenvironment promotes CCL28 levels to recruit Treg cells. In earlier reports, CCR10-expressing CD25+CD4+FOXP3+ Treg cells were identified to be critical for maintaining the potent anti-inflammatory properties in liver inflammation [10]. Furthermore, increased frequency of regulatory T cells has been detected in hepatocellular carcinoma (HCC) patients [22]. These observations are indicative of the possibility that the CD25+CD4+FOXP3+ Treg cells are required to suppress effective antitumor immune responses, and their migration to the tumor sites may be mediated by interaction with the CCR10 ligand CCL28 [23–25]. Our results are in accordance with the previous ones in that the tumor-infiltrated lymphocytes isolated from the liver tumor models have high levels of FOXP3. Considering CCL28 attracted the CD25+CD4+FOXP3+ Treg cell migration in a CCL28- and CCR10-dependent manner, it is conceivable that CCL28 directly binds and enriches the regulatory T-cells to promote liver tumor growth. Indeed, *in vivo* bioluminescence imaging results show that the fluorescent signals and weights of the CCL28-Hepa1-6-luci tumors seeded subcutaneously in a mouse were significantly greater than the Control-Hepa1-6-luci ones. More data, including direct quantification of the *in vivo* CD25+CD4+FOXP3+ Treg population and evaluation of the effects of immunoblocking with specific CCL28 antibodies on the *in vivo* tumor growth, are required to obtain from the mouse hepatic tumor model generated in this study, and will convincingly establish the repressive role of Treg cells in maintaining antitumor immunoresponse during liver tumorigenesis. On the other hand, CCL28 can also promote survival, proliferation and migration of primitive hematopoietic cells [26, 27], suggesting the functions of CCL28 may be versatile. Given the similar mechanism of CCL28 in promoting ovarian and liver cancer development, it is necessary to distinguish the different molecular details mediated by CCL28 functions in different systems.

The molecular targets of HIF1α cover a wide range of cellular process [28, 29]. During cancer development, the tumor cells must overcome the growth barrier – the hypoxic microenvironment that is accompanied by rapid proliferation. For example, in basal-like breast cancers, hypoxia is associated with the recruitment of the regulatory T cells [30]. For cancers, enhanced angiogenesis may be a viable solution to this hurdle. In line with previous reports, our study confirms that hypoxia directly induces the upregulation of CCL28 in liver cancer. It remains to be elucidated, however, that how CCL28-mediated Treg-recruitment can promote tumor growth. So far, two mechanisms have been proposed for the Treg cells to stimulate tumor angiogenesis [31]. Treg cells may repress tumor-reactive T cells to promote angiogenesis [32], or they may directly contribute to the production of vascular endothelial growth factors (VEGFs) in the tumor microenvironment [11]. Although our current data have suggested the recruitment of the Treg cells to the tumor sites, the predominant mechanism by which the Treg cells regulate the tumor growth is still awaiting further studies in liver cancer. It is also likely that both mechanisms may contribute to liver tumorigenesis under hypoxia.

In summary, our study has demonstrated the regulation of chemokines on tumor growth within the hypoxic microenvironment in liver cancer. Specifically, hypoxia may stimulate the expression of chemokine CCL28, which in turn plays a major role in recruiting regulatory T cells to the tumor site and accelerating tumor growth. The proposed regulatory mechanism is significant for identifying new therapeutic targets for immunotherapy of liver cancers, and will help to solve the challenges imposed by pro-tumor immune response in cancer treatment.

## MATERIALS AND METHODS

### Isolation of Treg cells and transwell migration assay of CCL28

Human CD4+CD25+CD127DIM cells were prepared using the CD4+CD25+CD127DIM T-Cell Kit (Miltenyi Biotec, Inc., Germany) according to the manufacturer's protocol. The transwell assay was performed as the following: (1) Normoxic group (upper chamber – Treg cells, lower chamber – normoxic medium); (2) Hypoxic group (upper chamber – Treg cells, lower chamber – hypoxic medium); (3) CCL28 antibody group (upper chamber – Treg cells, lower chamber – hypoxic medium + CCL28 antibody [50 μg/mL]); and (4) CCR10 antibody group (upper chamber – Treg cells + CCR10 antibody [2.5 μg/mL], lower chamber – hypoxic medium). The assay was allowed to proceed for 4 h before cell counting by flow cytometry.

### Cell culture

Human haptic cell lines, including HepG2, Hep3B, and SK-Hep-1 were cultured in Dulbecco's Modified Eagle Medium (DMEM) supplemented with 10% fetal bovine serum (FBS) and incubated in 37°C and 5% CO_2_. When grown to 60% confluency, the cells were incubated either in normoxic (21% O_2_, 5% CO_2_) or hypoxic conditions (1% O_2_, 5% CO_2_). Cells were collected at 0, 8, 24, and 48 h. Supernatants from the cell cultures from different time points were filtered through 0.25 filters, aliquoted, and stored in −80°C. One part of the filtered supernatants was subject to ELISA test for CCL28 levels, and the other was reserved as conditional media for subsequent cell biology studies. The cells were subject to total mRNA extraction by TRIzol.

### Stable overexpression of CCL28 in Hepa1-6 cells

The CCL28-overexpression plasmid CCL28-pLV-luci was prepared by Sangon Biotech (Shanghai, China). Briefly, the EcoRI/BamHI-digested fragment of CCL28 cDNA was cloned into pUC19. The resultant vector was double-digested by the same enzymes, and ligated to the same digested pLV-luci. The final overexpression plasmid was named after CCL28-pLV-luci. Mouse hepatic cancer cell lines Hepa1-6 were subject to transfection with lentiviruses packaged by CCL28-pLV-luci

### Real time qPCR

The total mRNA was extracted by TRIzol according to the manufacturer's protocol, and 0.2 μg of mRNA was used as template for the reverse transcriptional PCR reaction (25 μL) which includes the following components: M-MLV 5×buffer (5 μL), 10 mmol/L dNTPs (1.25 μL), and M-MLV (1 μL). The reaction was incubated in 42°C for 1 h, followed by deactivation of the reverse transcriptase at 70°C for 10 min. Real time PCR was performed in 20 μL with 1× SYBR Select Master Mix and 100 nM of CCL28 primers. The PCR cycles were set as 95°C for 2 min, followed by 45 cycles of [95°C for 15 s, 60°C for 45 s]. The sequences of CCL28 primers are: sense 5′-AAGGAAATGTTTGCCACAGG-3′ and antisense 5′-ATGGCCGTATGTTTCGTGTT-3′ for human, 5′-ACCTTCATCGGAAACTCCAAAG-3′ and 5′-CTGTTAGGCTGGGAAAAGTTAGG-3′ for mouse. Quantification of the real time PCR results was performed with ΔΔCt method using β-actin as the reference gene.

### ELISA

Cell culture supernatants from different time points were measured for the protein levels of CCL28 and VEGF by ELISA (Thermo Labsystems, USA) following the manufacturer's instruction. The values were expressed as mean ± SD.

### Knockdown of HIF1α in hepatic cell lines

HIF1α was knocked down in HCC cells by lentiviral transfection using pLV-siHIF1α. The knockdown strains were cultured in hypoxic condition (1% O_2_, 5% CO_2_) for 72 h. The cells and medium supernatants were then harvested for experiments indicated in this study. Real time PCR of CCL28 mRNA and ELISA of CCL28 in supernatant were performed as described above.

### Xenograft mouse model for liver cancer

Mouse hepatic cancer cells overexpressing CCL28 (CCL28-Hepa1-6-luci) were grown to log-phase. The same cancer cells overexpressing empty plasmid DNA (Control-Hepa1-6-luci) were used as controls. For each mouse, a total of 5×106 cells were harvested and resuspended in 200 μL of cold PBS, followed by subcutaneous injection at left flank. In separate experiments, CCL28-Hepa1-6-luci and Control-Hepa1-6-luci were injected subcutaneously around right- and left-tibia, respectively, in the same mouse. *In vivo* imaging study was performed on IVIS imaging system. Tumors were collected 4 w after tumor cell implantation.

### Quantification of Foxp3 mRNA in tumor-infiltrating lymphocytes from xenograft tumors of liver cancer

Xenograft tumors were isolated from mouse, digested, and minced in petri dishes. A specific isolation solution (Tianjin Hao-Yang Biological Manufacture Co., China) was used for isolate the tumor-infiltrating lymphocytes from the homogenized tumor tissues. Total mRNA was extracted as described above, followed by real time transcriptional PCR for measuring Foxp3 mRNA levels. The Foxp3 primer sequences are: sense 5′-TGAGAAAGGCAAGGCCCAGTGC-3′ and antisense 5′-TGGTGGCTACGATGCAGCAAGA- 3′.

### Immunohistochemistry

Immunohistochemistry analysis was performed as described previously [33]. Murine tumors were fixed in 10% neutral buffered formalin for 24 hours. Then, tumors were processed for paraffin embedding. Of note, 5 mmol/L sections were used for hematoxylin and eosin staining and immunohistochemistry. Unstained sections were deparaffinized, rehydrated and stained for Foxp3 (Abcam, ab20034), VEGFa (Abcam, ab51745), CCL28 (Santa Cruz, sc-27341) and CD31 (BD Pharmingen, 558737).

### Protein extraction and western blot analysis

Whole-cell protein and tissue protein extraction was isolated as described previously [33]. Western blotting were performed as previously described [33]. Antibody binding was revealed using an HRP-conjugated anti-rabbit IgG or anti-mouse IgG (Sigma). Antibody complexes were detected using Immobilon Western Chemiluminescent HRP Substrate (Millopore) and exposure to X-Omat film (Kodak). Anti-hCCL28, anti-mCCL28 (Santa Cruz, sc-27341), anti-mVEGFa (Abcam, ab51745), anti-hHIF1α (Abcam, ab92498), and anti-mHIF1α (Abcam, ab187524).

### Flow cytometry

The following murine and human antibodies were used: anti–hCD4-FITC, anti–hCD25-APC, anti–hFOXP3- PE, anti–mCD4-FITC, anti–mCD25-APC, and anti– mFOXP3-PE (eBiosciences). Flow cytometry was carried out on the BD Accuri C6 PLUS flow cytometer (Becton Dickinson). Data were analyzed using FlowJo software.
